# Fine-grained changes to implicit memory for language processing across adulthood

**DOI:** 10.3389/fpsyg.2026.1800437

**Published:** 2026-05-11

**Authors:** Willem S. van Boxtel, Ashley Hart, Zoey Gautreau

**Affiliations:** Department of Communication Sciences and Disorders, Louisiana State University, Baton Rouge, LA, United States

**Keywords:** cognitive aging, explicit memory, implicit memory, sentence processing, structural priming

## Abstract

**Background:**

Traditionally, age-related changes to implicit memory have been considered minor or non-existent, especially when compared to changes to explicit cognition. Similarly, linguistic tasks relying on explicit abilities frequently show substantial age-related impairments, while implicit measures do not. Whether this dichotomy is ecologically valid, and whether fine-grained changes to implicit memory occur across the adult lifespan, nevertheless remains unclear. This study aimed to investigate implicit memory functioning in an age-continuous sample and relate implicit memory to language processing.

**Methods:**

A large age-continuous sample (*n* = 148) completed a set of explicit and implicit cognitive tasks followed by a structural priming task. Age-related changes to cognitive and linguistic task performance were assessed, and individual cognitive scores were related to linguistic effects.

**Results:**

Greater age was associated with lower explicit but not implicit cognitive performance in line with past research. Close inspection nevertheless revealed reliable age-related changes on some— though not all—aspects of implicit memory. Linguistic performance remained stable with age, and was unaffected by either implicit or explicit memory scores.

**Conclusions:**

This study supports the notion that fine-grained changes to some aspects of implicit memory result from aging. However, no changes to linguistic processing were found and performance was unrelated to memory scores, suggesting older adults either have intact linguistic processing or successful compensation mechanisms for language.

## 1 Introduction

One of the primary cognitive challenges facing older adults is a general decline in memory functioning (Bäckman et al., 2001; Nyberg and Pudas, 2019; Salthouse, 2003). As the world ages to an extent never before seen in human history (Harper, 2014; United Nations, 2024), larger components of the population will face memory-related issues, making a thorough understanding of the implications of such issues for human functioning absolutely critical. Research on memory and cognitive aging has traditionally distinguished between explicit, declarative, or conscious memory on the one hand, and implicit, unconscious memory or learning on the other; while explicit memory shows marked declines in older age (e.g., Park and Festini, 2017; Bäckman et al., 2001), implicit functions may be relatively stable across adulthood (see Reber et al., 1999; Reber and Allen, 2000; Rieckmann and Bäckman, 2009, for reviews). This view has nevertheless been challenged in some work (e.g., Maybery et al., 1995; Simon et al., 2011; see Howard and Howard, 2022, for a review), and it is yet unclear what specific implicit functions decline with age.

Memory is intricately tied to language processing, yet it is unclear which facets of the language processing system—if any—show age-related declines, and to what extent changing implicit memory capacities underpin such declines. While explicit memory deficits have been tied to poorer performance on a variety of language tasks by several existing theories (Just and Carpenter, 1992; Fedorenko et al., 2006; Martin, 2021), linguistic processing appears to involve the engagement of both implicit and explicit memory (e.g., Martin, 1993; Lewis et al., 2006; Conway and Pisoni, 2008; Hodapp and Rabovsky, 2021), and the relative importance of both systems for sentence processing is not well-understood. In particular, the relation between possible changes in implicit memory in older populations and sentence-level processing remains unclear. Performance on sentence processing tasks that primarily tap implicit memory, such as syntactic priming, appears to remain largely stable across the lifespan (Hardy et al., 2017, 2020; Van Boxtel and Lawyer, 2023a,b; Weirick and Lee, 2024), yet it has proven problematic to relate such performance to tests of implicit memory (e.g., Man, 2023).

Whether core components of implicit memory and learning indeed decline with age, and whether such declines affect linguistic processing, is therefore still uncertain. Addressing these isues is nevertheless highly critical, as cognitive-linguistic functioning in an increasingly larger cohort of older adults in society will affect how governments, companies, and other stakeholders adapt to the needs of a greater aging population. Further, given that the incidence of acquired neurogenic disorders is rapidly increasing, and that these disorders are often paired with impairments to memory (Mayer and Murray, 2012; Minkina et al., 2017), understanding how different components of memory contribute to linguistic processing will be essential if effective treatments and assessments for such disorders are to be developed.

The current study sought to establish whether implicit memory and learning processes change across an age-continuous sample and whether the contribution of implicit memory to sentence processing changes concurrently. A cognitive battery including multiple explicit and implicit memory tasks, as well as a structural priming task were administered. Participant-level memory scores were related to the structural priming task, and fine-grained analyses of individual memory task performance are also reported.

### 1.1 Implicit memory and aging

Cognition changes drastically as humans age. While many physical and cognitive faculties undergo marked changes later in life, declines in memory functioning are possibly the most recognized and impactful age-related issue (e.g., Small, 2001; Bäckman et al., 2001; Luo and Craik, 2008; Stephan et al., 2016). Findings from neuroimaging suggest such memory declines are symptomatic of reduced functional connectivity between cortical and sub-cortical areas critical to memory (such as the hippocampus, e.g., Nyberg, 2017) or of reductions in general neural network coherence (Beason-Held et al., 2017). Regardless of neurphysiological causes, memory declines have crucial social impact (Parikh et al., 2016) and may be indicative of clinical (i.e., non-typical) cognitive declines later in the aging process (Buckley et al., 2016; Warren et al., 2022).

However, memory is a multifaceted component of cognition, and not all aspects of memory decline in the same way—if they decline at all. In particular, the dissociation between explicit or declarative memory and implicit or non-declarative memory (or learning) has received substantial attention in the context of cognitive aging (see Park and Festini, 2017; Bäckman et al., 2001, for reviews). For the purposes of this study, we consider the terms ‘implicit learning' and ‘implicit memory' roughly equivalent, though not disregarding possible nuances and past theoretical work on distinguishing specific implicit cognitive functions (e.g., Reber, 2013; Han et al., 2022). Indeed, we acknowledge that some frequently used implicit tasks may be reflective of at least somewhat distinct implicit functions which relate to conscious, explicit functions to varying degrees; for instance, perceptual identification tasks may be more closely tied to more overt, explicit cognition (see, e.g., Janacsek and Nemeth, 2013; Mazancieux et al., 2020, for discussions). Similarly, while we use ‘explicit memory' to refer to any memory-related function that requires conscious recall or learning operations (encompassing short-term memory, working memory, and related memory types; see Baddeley, 1995, 2020; Squire and Dede, 2015), we acknowledge that it may be more accurate to view implicit and explicit memory as a continuum of partially overlapping cognitive functions (e.g., Kihlstrom, 1993; Erdelyi, 2012). While a full theoretical discussion of this graded nuance is beyond the scope of this paper (see, e.g., Reber, 1996, for theoretical accounts), we do not view implicit and explicit memory as purely isolated, non-overlapping components of cognition. However, for the purposes of assessing both aspects of memory separately, we maintain the distinction that explicit memory involves an active search through short or long-term memory systems, and implicit memory involves the unconscious, unaware adaptation of cognitive parameters to incoming input (Schacter et al., 2014; Dew and Cabeza, 2011).

Age-related impairments to implicit memory are far less well-documented than those in explicit memory. Implicit memory impairments may be virtually non-existent, or may be more dependent on specific task demands (see Rieckmann and Bäckman, 2009, for a review). However, this may not be sufficient to explain some findings of implicit age-related declines. For instance, (Howard and Howard 2022) review studies of sequence learning in older adults [e.g., using a Serial Reaction Time (SRT) task, in which participants learn a repeating sequence of trials], suggesting greater age is associated with gradual inefficiencies to the sequence learning system that extend beyond mere overall cognitive slowing (see Salthouse, 1996). These deficits may be especially pronounced when sequence learning must be probabilistic—that is, if a sequence does not *always* predict a future sequence (see Howard and Howard, 2013, for a review). Importantly, these declines are thought to be gradual: there is no single cut-off age at which implicit memory becomes inefficient. Rather, inefficiencies are shown gradually across the adult lifespan (Howard and Howard, 2022, 2013).

Alternatively, while some components of implicit memory may show age-related inefficiencies, as (Howard and Howard 2022) and others suggest (but with which others disagree, e.g., Rieckmann and Bäckman, 2009), older adults' effective compensation mechanisms may allow for generally intact behavioral performance during many activities that require implicit memory deployment (Woodruff-Pak and Hanson, 2013; De Frias et al., 2003; Radnan et al., 2023). Indeed, overarching cognitive slowing in older age has been considered a compensation mechanism in itself by some past authors (e.g., Stine-Morrow et al., 2006; Lemaire, 2010; though cf. Malyutina et al., 2018). This may be especially relevant for language processing, which is a largely implicit set of operations that must occur rapidly without much conscious awareness (Van Gompel, 2013). Performatively, language nevertheless seems to remain relatively stable in older adulthood (see Van Boxtel and Lawyer, 2021, for a review). We discuss the interplay of language and memory in more detail below.

### 1.2 Implicit and explicit memory for language processing

Linguistic processing involves the decoding and interpretation of linguistic utterances into a single, comprehensive meaning (Frazier, 2016; Cooper, 2013). While many different theories of linguistic and sentence processing exist, most accounts agree that the full understanding of utterances involves grammatical, lexical, and discourse information being combined into a single representation (Van Gompel, 2013). Such processing is therefore a highly complex operation, which requires the use and co-ordination of multiple linguistic, meta-linguistic, and cognitive faculties, including attention (Mishra, 2013), inhibitory control (De Rom et al., 2023), and—crucially—memory. In particular, the role of short-term memory (STM) and working memory (WM) have been highlighted in past studies. These types of memory involve the short term storage, maintenance, and subsequent retrieval of information (Baddeley, 2020; Jonides et al., 2008), which has been hypothesized to be crucial for the correct selection of syntactic dependencies, referents, and semantic information (Gupta, 1996; Gathercole and Baddeley, 2014).

Performance on linguistic tasks has been tied directly to STM and WM capacity, and specific language-based memory tasks (such as the Reading Span Task, see Daneman and Carpenter, 1980; Daneman and Hannon, 2007) have been developed and used as predictors of sentence processing ability. These tasks are generally very explicit: for instance, Reading Span tasks require participants to make judgments about the plausibility of presented sentences, and simultaneously remember the final word of each sentence. These sentences are presented in increasingly longer sets, and participants are asked to recall the final words of each sentence after each set. Other tasks employing different versions of the same set-up include operation span tasks (Unsworth et al., 2005) and *n*-back tasks (Jaeggi et al., 2010; for an overview of span tasks, see Conway et al., 2005).

Consequently, it is perhaps unsurprising that past researchers have emphasized how language processing, especially at the sentence level, declines with age. Given that several types of memory appear to decline with age, and that memory is crucial to language processing, it would naturally follow that language processing becomes less effective as people age (see Van Boxtel and Lawyer, 2021, for a review). Indeed, early studies offered evidence for this notion: for instance, (Obler et al. 1991) found that older adults were significantly slower and less accurate than younger adults on acceptability judgments about complex syntactic structures. This type of evidence has been widely interpreted as offering evidence for the decline of language processing in older adults (e.g., by Grossman et al., 2002; Johnson, 2003; Poulisse et al., 2019). However, the few existing studies using implicit, non-declarative paradigms for assessing language in aging have found little or no age-related declines or differences (Federmeier et al., 2003; Van Boxtel and Lawyer, 2023a,b). Other studies have found age effects on more explicit measures which subsequently disappear when performance on cognitive tasks and/or measures of working memory are taken into account (e.g., DeDe et al., 2004). Finally, some authors have theorized that, rather than representing cognitive inefficiency or linguistic decline, the longer response times often shown by older compared to younger groups to linguistic tasks are a result of older adults' more expansive vocabularies, which necessitate a longer mental lexicon search (e.g., Ramscar et al., 2014). There is, therefore, still significant controversy over whether language processing declines with age, or whether the measurement of meta-linguistic declines has been interpreted as a language-specific issue.

### 1.3 The present study

In short, this study sought to address several open areas of inquiry related to possible declines of implicit memory in older adults, and how such declines relate to language processing. Specifically, we aimed to answer the following questions:

1. Do fine-grained implicit memory operations, as indexed by two different tasks, decline across the adult lifespan, and if so, does this decline mirror age-related impairments to explicit memory?
2. How does implicit memory relate to language processing across the lifespan, and do older adults rely on intact implicit memory abilities to process language successfully?

We made the following hypotheses around each question. First, we predicted that age-related declines on implicit memory would be far less substantial than those observed in explicit memory tasks, if they exist at all. This decline should be gradual across the lifespan. Second, we predicted little to no behavioral changes to language processing across the lifespan, but made no specific hypotheses about the reliance of older adults on implicit memory during language processing.

## 2 Materials and methods

### 2.1 Cognitive tasks

#### 2.1.1 Participants

**Figure 1:**
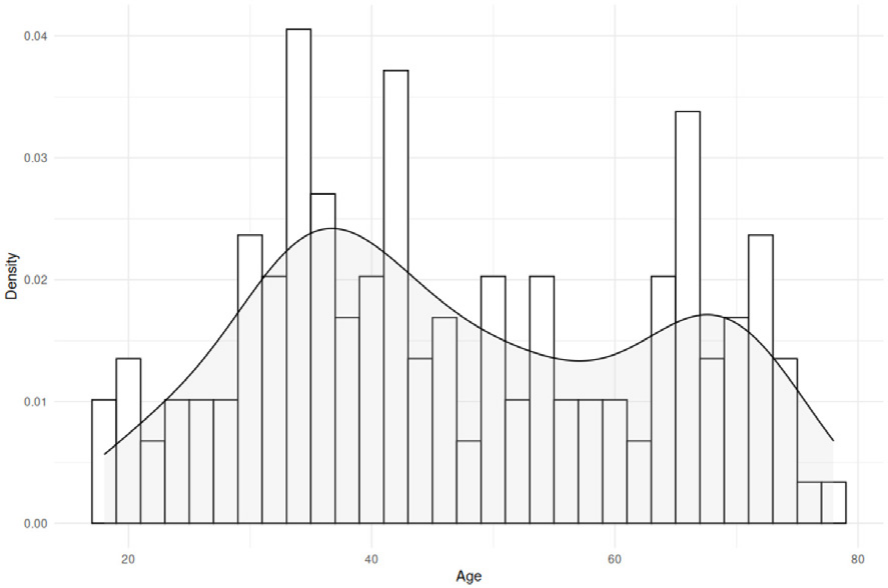
Histogram showing the age range of participants included in this study. Each bar represents two years; the gray line represents frequency density.

One hundred and forty-eight participants took part in this study through the Prolific.com platform (Prolific, 2024). They were compensated for their participation; this study received full ethical approval from the local institutional review board. A *post-hoc* power analysis using the *pwr* package (Champely, 2020) in R 4.3.3 (R Core Team, 2024) showed that this study had good statistical power (0.808) with the included sample size, a significance level of α = 0.05, up to three predictors, and a small anticipated effect size (0.075). Participants' chronological ages ranged from 18 to 80 (*M* = 41.7, SD = 12.9), and ages were distributed such that there was good representation in both younger and older age ranges (see Figure 1 for a histogram). Participants self-reported they were dominant speakers of North American English and denied the presence of any neurological, cognitive, or linguistic impairments (e.g., stroke, Autism, ADHD, developmental language disorder). Fourteen additional participants took part in the experiment but were rejected either because they reported not being a dominant English speaker or the presence of a neurological/cognitive/linguistic impairment).

**Figure 2:**
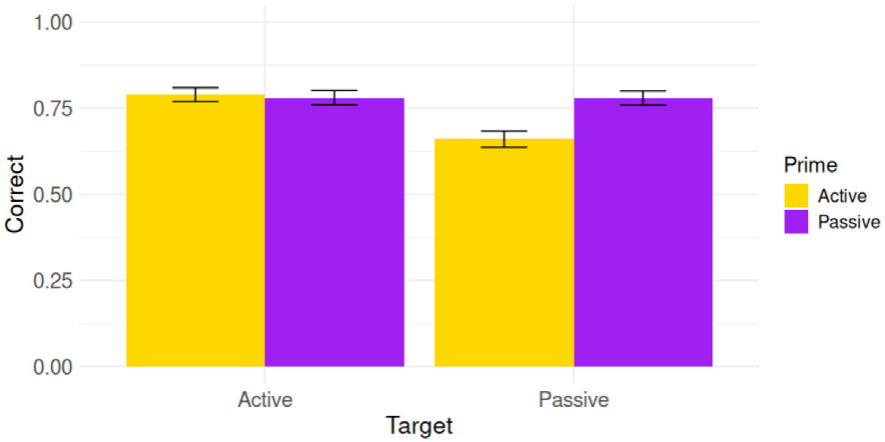
Accuracy on the structural priming sentence-picture matching task by prime and target structure, showing clear priming effects on passive targets, but possibly not on actives. Error bars represent standard error of the mean.

This study was run online using an online recruitment platform to allow for rapid, large-scale data collection of an age-continuous sample. Many online recruitment platforms have received substantial negative attention from behavioral researchers for high levels of fabrication and inattention (e.g., Roehl and Harland, 2022; Thompson et al., 2024); however, Prolific.com has received favorable evaluations from studies comparing platforms' data quality (e.g., Peer et al., 2022), and we constructed several safeguards to ensure sufficient data quality. First, participants could take part only on personal computers (NOT on tablets or phones) to ensure all participants saw the stimuli with sufficient resolution, and to improve attentiveness. Second, oral repetitions of prime sentences (see Section 2.2.3 for methodological detail) were checked manually by the second author, who ensured recordings were present, accurate, and understandable. Finally, participants who took a disproportionately short (< 30 min) or long (> 1.5 h) time to complete the main task were not included in our sample; and accuracy on the main priming task was substantially above chance (see Figure 2 below), suggesting the included participants did pay attention to the task.

Participants completed two tests of implicit memory, a visual SRT task and the Fragmented Picture Task (FPT), and three tests of explicit memory, the Picture Pointing Span task (PPS), Word Pointing Span task (WPS), and Reading Span Task (RST). Three rather than two explicit measures were included as the PPS and WPS take a highly similar format (see Section 2.1.4), and either task takes a short time (approximately 5 min) to complete; it was therefore practical to include both the picture (PPS) and written word (WPS) format of the test.

We used the SRT and FPT as our implicit measures given their frequent past use and past connections to structural priming literature (e.g., Schuchard and Thompson, 2014; Heyselaar et al., 2021). We nevertheless acknowledge that both tests may measure somewhat distinct implicit abilities: the SRT appears to reflect procedural sequence learning (Schuchard and Thompson, 2014), while the FPT may be seen to approach sequence identification (though see Kessels et al., 2011; Heyselaar et al., 2017, for discussions of the FPT's implicit nature). Both tasks nevertheless rely on non-overt, implicit learning of a stimuli sequence, and our main measure for the FPT (as described in Section 2.1.3 below) was a delayed adaptation to a previously learned sequence, reflecting processes closely related to the SRT. We therefore deemed our combination of SRT and FPT scores into a single composite score (see Section 2.3) to be justified.

Before commencing the tasks, participants provided informed consent and completed a biodata questionnaire. All participants completed the FPT first, as this task requires a separate follow-up round (see Section 2.1.3). Following the FPT, the order of the remaining tasks was counterbalanced, except that the FPT follow-up was presented after the task that followed the main FPT (thus, the overall sequence was FPT—Task 2—FPT follow up—Task 3—Task 4—Task 5). The order tasks was semi-random, such that sixteen different task sequences (consisting of PPS, WPS, RST, and SRT) were created; one in every sixteen participants followed each sequence. This semi-random structure was adopted to minimize fatigue effects on any one task while keeping the FPT and its follow-up task as true to the original design (see Section 2.1.3) as possible. All cognitive battery and language task materials are available from our online supplementary materials at https://osf.io/4mw5x.

#### 2.1.2 Serial Reaction Time task (SRT)

**Figure 3:**
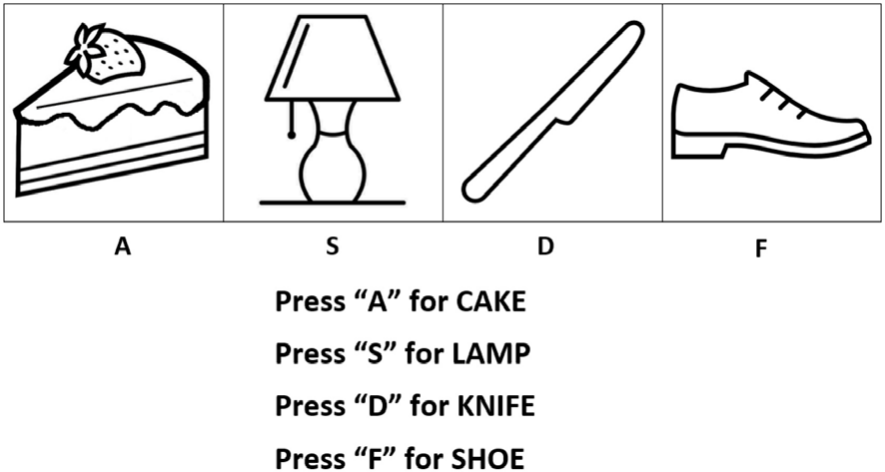
Example serial reaction time display. This display was paired with auditory recordings of a female native English speaker reading one of these four words.

The SRT used for this study was modeled after a version designed for individuals with aphasia by (Schuchard and Thompson 2014). Participants were presented with a list of eight auditory word-picture matching trials, each involving one of four words and pictures (cake, shoe, knife, lamp; see Figure 3 for an example). This sequence of eight trials was repeated 48 times to encourage participants to implicitly learn the sequence (for a total of 384 trials in the learning phase, equally separated into six blocks). Following this repetition, a seventh block of 64 completely random trials was presented, in which we anticipated significantly longer response times. Finally, block eight comprised the earlier sequence repeated a further eight times. The main measure of interest was the average difference in response times between blocks eight and seven, reflecting how effectively participants recognized the sequence they had learned earlier (known as the Rebound effect; see Schuchard and Thompson, 2014, for further reference). However, additional measures of learning can be extracted by gauging how much response time improvement occurred between block 1 and block 6. Four practice trials preceded the main part of the task; participants were encouraged to press the button corresponding to the word they heard as quickly and accurately as possible. Incorrect trials were removed from the data prior to any score computation or analysis.

#### 2.1.3 Fragmented Picture Task (FPT)

**Figure 4:**
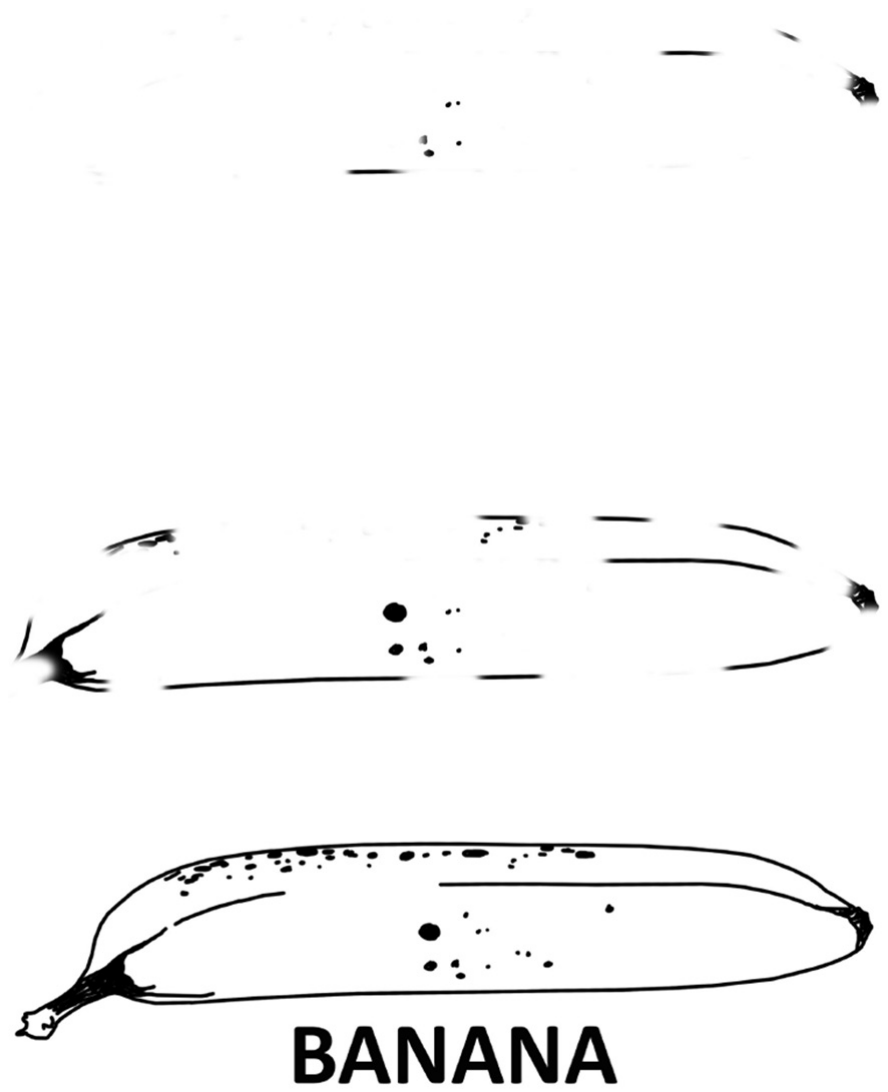
Fragmented picture task example, showing the first stage of fragmentation, the fourth stage, and the complete stage.

The FPT in this study (modeled after Kessels et al., 2011) used eight line drawings presented in decreasing stages of fragmentation, and asked participants to name each picture as soon as they recognized it and to press the space bar after each naming. Each fragmentation stage was presented for three seconds, and if participants did not name a picture a final stage was presented showing the complete picture and its written name. The sequence of eight pictures was repeated three times to encourage implicit learning of the sequence; following these three rounds, participants completed a different cognitive battery task, after which they returned to the FPT for a follow-up round. Audio recordings of participants were made and checked for accuracy, and to ensure that all participants actually named the pictures and did not simply press through the task. The main by-participant FPT measure comprised the difference between the average response stage during the first sequence and the fourth (follow-up) sequence. For example, a participant may have responded, on average, at fragmentation stage five in the first round, but at stage two in the fourth, giving them an FPT score of three. See Figure 4 for examples of fragmentation. Two practice trials were presented before the main task.

#### 2.1.4 Explicit memory tasks

Explicit memory was indexed using the Picture Pointing Span (PPS), Word Pointing Span (WPS), and Reading Span Tasks (RST). The PPS and WPS in this study were adapted from the Temple Assessment of Language and Short-Term Memory in Aphasia (TALSA; Martin et al., 2018). Both tasks involved participants listening to lists of words increasing in length from two to four words, then clicking on pictures (PPS) or written words (WPS) corresponding to the words they heard presented in a 3 × 3 grid. Each list length was presented four times, such that four two-word trials were presented, followed by four three-word trials, and so on. After the four-word trials finished, participants completed a second round of trials in which they recalled words in *reverse* order to what they heard. Thus, the PPS and WPS indexed both short-term and working memory. Participants' PPS and WPS scores were calculated as the total number of correct trials. No partial credit was awarded given the relatively easy nature of these tasks.

The RST for this study was adapted from (Van Boxtel and Lawyer 2023a). Participants read increasingly longer sets of sentences, judged whether each sentence made sense or not, and remembered the last word of each sentence. They recalled these words at the end of each set. Sets increased in length from two to eight sentences; half of sentences did not make sense (e.g., “I saw a monkey doing my taxes”) and the other did make sense (“We're hosting a barbecue this weekend”). Participants were awarded a full point if they recalled a word in the position in which it was presented (for a maximum score of 35 points), and a half point for words recalled in the correct set, but the wrong position (following Conway et al., 2005). Interpretable misspellings were accepted. Two two-sentence practice trials preceded the main task.

### 2.2 Structural priming task

#### 2.2.1 Participants

The same participants who completed the cognitive battery were invited to participate in a structural priming sentence-picture matching task to gauge linguistic processing. Of the 148 participants who completed the cognitive battery, 121 completed the priming task. Sixteen of these participants were rejected because their prime reading accuracy could not be verified (see below) or for technical reasons, resulting in a final sample size of 105 for this experiment. A power analysis showed that this sample could yield statistical power of 0.783 with three predictors, a significance level of α = 0.05, and considering a small effect size (0.1). Participants were 48.38 years old on average (SD = 15.36) with a range of 18–75 years.

#### 2.2.2 Materials

Stimuli for this task were adapted from (Van Boxtel et al. 2024, under review): 60 pairs of pictures predicting opposite transitive events were used (e.g., a butler pushing a nun, and a nun pushing a butler). Each pair of pictures was accompanied by an auditory sentence recording, depicting one of two pictures. The participants' task was to match the auditory recording to one of two pictures. In half of trials, the auditory recording was a passive sentence (e.g., “The butler is pushed by the nun”), while the other half contained an active sentence (e.g., “The nun is pushing the butler”). One picture in each pair therefore served as target, while the other served as a distractor image. Each picture pair was preceded by a prime item, consisting of oral repetition of a visually and auditorily presented prime sentence. This prime was an active sentence in half of trials, and a passive in the other half, resulting in a 2 × 2 design (active v. passive prime and active v. passive target). Following substantial prior research (e.g., Ferreira and Bock, 2006; Ledoux et al., 2007; Jaeger and Snider, 2013; Mahowald et al., 2016; Hardy et al., 2017, 2020; Leonard et al., 2022; Van Boxtel and Lawyer, 2023a,b) we anticipated faster, more accurate responses when primes and targets matched in syntactic structure compared to when they did not. This structural priming paradigm is thought to reflect essential aspects of language processing, including communicative adaptation to interlocutors, efficient transfer of information, and—critically—implicit learning of grammar (see Ferreira and Bock, 2006, for a discussion).

**Figure 5:**
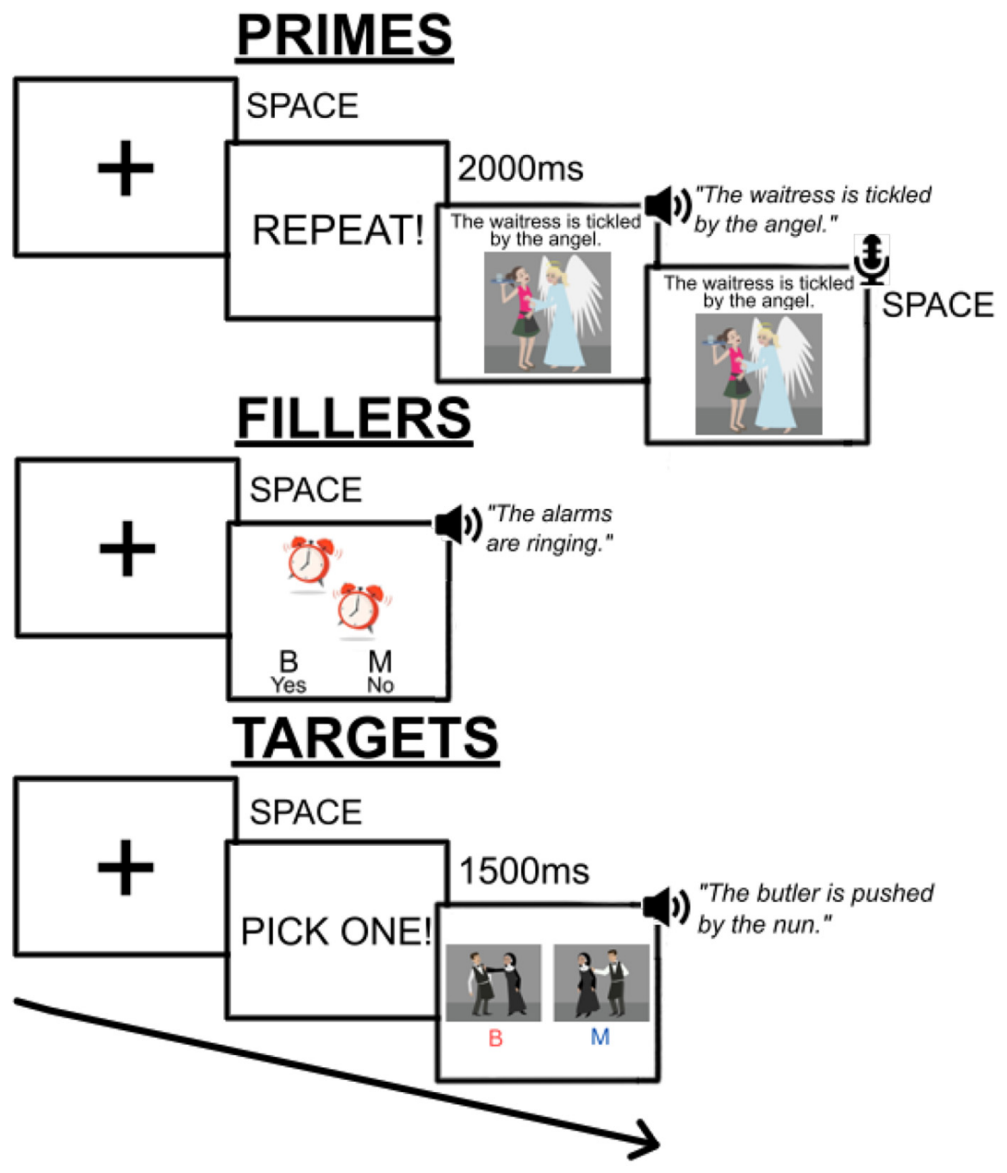
Schematic overview of the sentence-picture matching task, showing a passive prime trial.

In target sentences, each verb was used a maximum of four times across structures, and the same nouns did not appear more than four times in the same position (agent, i.e., “the nun” in the previous example; and patient, i.e., “the butler”). As detailed in text footnote 1, noun length and frequency was matched in each structure, such that the first and second nouns in both passives and actives were not significantly more or less frequent, or longer or shorter. The full list of sentences appears in our supplementary materials at https://osf.io/4mw5x. See Figure 5 for examples of prime and target displays.

In addition, 120 filler sentences and pictures were used; these fillers comprised intransitive declarative structures (e.g., “The alarms are ringing”) and took an auditory sentence-picture verification format, requiring participants to select whether the sentence they heard matched the picture they saw. Half of fillers presented non-matching pictures and sentences, requiring a negative response, while the other half presented matching pictures and sentences. All sentences, fillers and critical trials, were recorded by a female native speaker of North American English, and volume was equalized at conversational level.

#### 2.2.3 Procedure

See Figure 5 for a schematic overview of the task. The task consisted of 60 prime-filler-target-filler sequences. The task was coded and presented using Gorilla.sc (Cauldron Science Ltd, Newbury, United Kingdom) (Anwyl-Irvine et al., 2020), and three practice trials consisting only of filler sentences were presented prior to the main task. Each prime consisted of a fixation cross which was terminated by the participants pressing the space bar. This was followed by a 2,000 ms reminder that they should repeat the sentence they heard and saw. Each prime played in full, following which a monotone beep sound was presented, indicating that the participant could start their repetition. They ended their repetition by pressing the space bar. Accuracy of repetition was checked prior to data analysis (see Section 2.3). Following each prime, a filler sentence was presented as a sentence-picture verification trial; participants pressed B or M to indicate whether the picture they saw matched the sentence they heard. Target sentences began with another fixation cross, followed by a 1500ms reminder that they should pick one of two pictures. Both pictures were presented and the audio began playing simultaneously. Participants could not respond until the audio finished playing completely; they again pressed B or M to select which picture they thought they heard.

### 2.3 Analysis

As discussed above, the main measure of interest in the SRT was the Rebound effect, that is, the difference between response times in Block 7 (random block following 6 sequential patterned blocks) and Block 8 (return to sequential patterned block from blocks 1-6). This measure was computed for each participant individually for correlation with language task performance and for computation of a composite score of implicit memory. However, we also assessed performance on the SRT in a more fine-grained manner by evaluating all response times for all participants by block and chronological age. To this end, we built linear mixed-effects regression models using the *lme4* library (Bates et al., 2015) running in R 4.4.4 (R Core Team, 2024). These models included a fixed interaction of block * age, and random intercepts of participant and item (random slopes of participant by item were attempted, but resulted in model convergence issues). Response time data were trimmed to remove incorrect responses as well as outliers: values above or below 2.5 standard deviations from each participant's individual mean were removed. Further, response times were log-transformed if normality of model residuals was improved by such transformation. Model evaluation was achieved in the *lmerTest* (Kuznetsova et al., 2017) and *emmeans* libraries (Lenth and Piaskowski, 2025).

The FPT was analyzed similarly to the SRT. By-participant FTP scores were computed by deducting the participant's average response stage in the fourth repetition of the FPT from their average response stage in the first presentation. This metric indexed the implicit sequential learning of FPT stimuli. Additionally, we modeled the complete FPT response time data by an interaction between repetition and age in a linear mixed-effects model, to more carefully evaluate how age affected FPT performance. The same model building libraries and strategies as used in the SRT data were employed for the FPT.

The FPT and SRT on the one hand, and the WPS, PPS, and RST on the other hand, were combined into composite scores of implicit and explicit memory by *z*-transforming and averaging each by-participant score. Thus, composite implicit scores (IS) were calculated as *IS* = (*z*(*SRT*)+*z*(*FPT*))/2 while composite explicit scores (ES) were calculated as *ES* = (*z*(*WPS*)+*z*(*PPS*)+*z*(*RST*))/3). While computing such composite scores can cause statistical confounds, we deemed the constituent parts of our composite scores to be sufficiently related, and measuring similar constructs, to justify inclusion in the composite scores. Both the SRT and FPT measure implicit adaptation to incoming stimuli, and all three explicit measures indexed conscious recall of previously processed information.

Prior to analysis of the priming task, we checked prime repetition accuracy for all participants by listening to and verifying their prime reading responses. This led to the exclusion of the 16 participants mentioned earlier who either did not repeat primes or for whom prime reading could not be verified due to technical issues. While both accuracy and response times were yielded by the priming task, only accuracy results are reported here as no effects were apparent on the response time data anywhere. Predictor variables for this analysis included the implicit and explicit composite memory scores from the cognitive tests, age, and priming condition. We defined ‘primed' trials as active targets preceded by active primes and passive targets preceded by passive primes, and assigned an ‘unprimed' label to targets with non-matching primes. Unprimed trials were treated as the reference level for models reported in the results. Past priming studies have found reliable *inverse frequency effects*, that is, greater priming effects in less frequent structures, such as passives (see Mahowald et al., 2016; Zhou, 2021). We therefore also considered passive targets separately in our analyses. Accuracy on the priming task was analyzed with generalized linear mixed-effects models with a binomial distribution, including fixed-effects interactions of age, implicit memory score, and explicit memory score. Models were built with a maximum random effects structure as far as convergence allowed. We began with random slopes of participant by trial, resorting to random intercepts of participant and trial when necessary. The most complex random effects structure (including random slopes) was always attempted first, while maintaining the required fixed effects interactions, before we resorted to random intercept structures instead. When random slope models failed to converge, we included random intercepts of participant and trial, and if these models also failed to converge, we attempted a random intercept of participant alone (though this was not the case for any models reported in this paper). Each model table reports details on the model's random effects structure. We expected primed trials to yield higher accuracy than unprimed trials, but made no specific hypotheses regarding effects of age or implicit or explicit memory.

Finally, where theoretically relevant null results occurred, we confirmed the absence of effects using Bayesian mixed-effects model comparisons using the *brms* (Bürkner, 2021) and *bridgesampling* packages in R (Gronau et al., 2020). These analyses contrasted a full model (including the effect or interaction of interest) with a null model containing all random and fixed effects *except* the effect or interaction of interest. All Bayesian models were fitted with weakly informative Gaussian priors. Where possible, the same random effects included in the main frequentist models were also included in Bayesian models. Following (Lee and Wagenmakers 2013), we interpreted the Bayes' Factors (BFs) yielded by this analysis as supporting the null model when under 1, as ambivalent when between 1 and 3, and as supporting the full model when over 3. Our full analysis pipeline including Bayesian models can be viewed in the supplementary materials at https://osf.io/4mw5x.

## 3 Results

### 3.1 Memory aging

**Figure 6:**
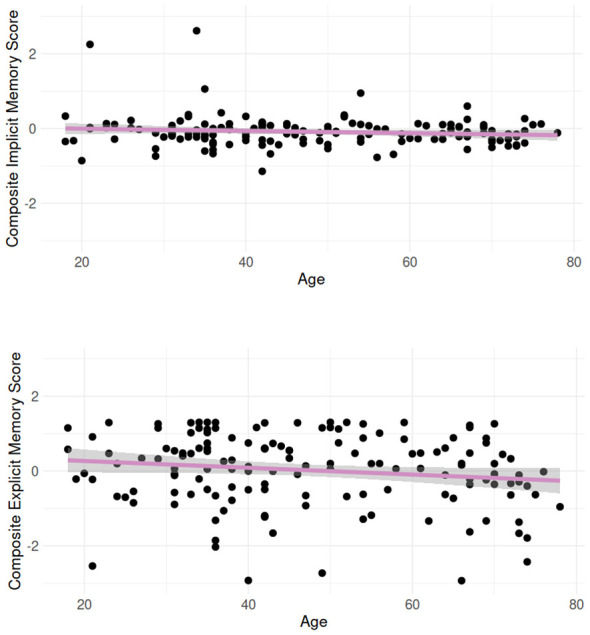
By-participant composite scores of implicit memory (above) and explicit memory (below), showing reliable (though minor) declines of explicit scores by age, but no age-related declines on implicit measures.

**Figure 7:**
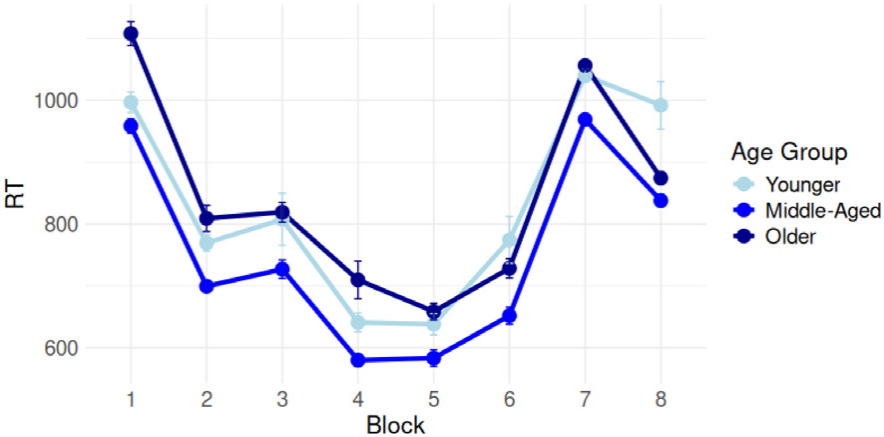
Performance on the Serial Reaction Time task (SRT) in three equally split age groups, showing gradual learning of the repeated sequence before drastic increases in response times when the repeated sequence was interrupted. The difference between blocks 7 and 8 consituted our SRT Rebound measure.

**Figure 8:**
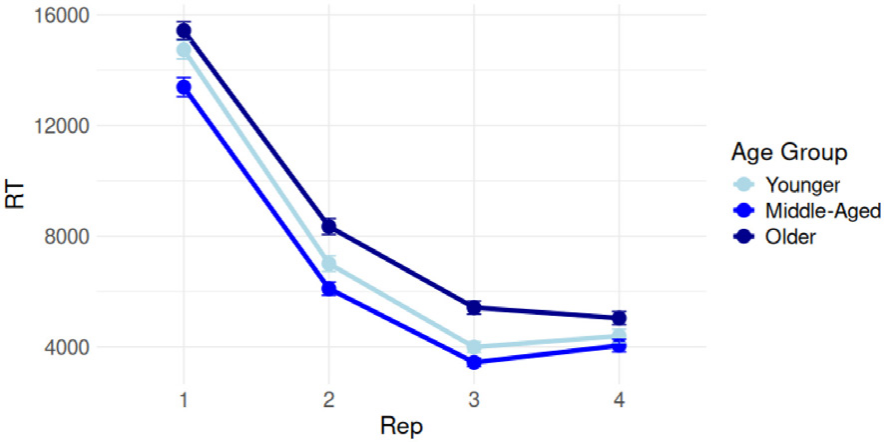
Performance on the Fragmented Picture Test (FPT) in three equally split age groups, showing gradual learning of the stimuli between repetitions 1 and 4. The difference between Reps 1 and 4 consituted our main by-participant FPT measure.

Overall, by-participant composite implicit scores (IS) and composite explicit scores (ES) were assessed for correlations with age. IS did not correlate with age (*r* = –0.156, *p* > 0.05), while ES correlated significantly (though weakly) with age (*r* = –0.192, *p* < 0.05). This is suggestive of age-related declines to explicit, but not implicit memory functioning. Additionally, IS and ES were not correlated (*r* = –0.035, *p* > 0.05), suggesting both measures tapped different cognitive processes. Visualizations of IS and ES by age are given in Figure 6. We expanded on this analysis with a more fine-grained evaluation of learning performance on the SRT and FPT by age. Figure 7 shows response times on the SRT by three equally split age groups (these groups are used only for visualization purposes; age was treated continuously in all analyses), while Figure 8 shows a similar visualization for the FPT. We first modeled SRT response times in the first five blocks by age and block (we excluded block 6 as RTs unexpectedly increased in this block, see Figure 7). This model is summarized in Table 1. FPT response times were modeled similarly (summarized in Table 2) but included all repetition stages.

**Table 1:** Model summary of Serial Reaction Time task (SRT) model assessing sequence learning in the first five SRT blocks (*logRT*~Block*Age).

Parameter	Est.	SE	*t*	*p*	*OR*	*95% CI*
(Intercept)	6.308	0.1102				6.092; 6.524
Block 1 to 2	0.075	0.038	1.985	**0.048**	1.078	0.001; 0.148
Block 1 to 3	0.008	0.037	0.207	0.836	1.008	–0.065; 0.081
Block 1 to 4	–0.216	0.037	–5.842	<**0.001**	0.806	–0.288; –0.143
Block 1 to 5	–0.314	0.037	–8.465	<**0.001**	0.738	–0.386; –0.241
Age	0.003	0.022	1.170	0.244	1.002	–0.002; 0.007
Block 1 to 2 * Age	–0.001	0.000	–3.036	**0.002**	0.999	–0.002; –0.000
Block 1 to 3 * Age	0.000	0.000	0.246	0.806	1.000	–0.001; 0.001
Block 1 to 4 * Age	0.001	0.000	2.408	**0.016**	1.000	0.000; 0.002
Block 1 to 5 * Age	0.001	0.000	3.050	**0.002**	1.000	0.000; 0.002

Blocks were contrast coded such that each block was compared to block 1. Values meeting the significance threshold of α < 0.05 are marked in **bold**. This model included random intercepts for participant (σ = 0.181, SD = 0.425) and item (σ = 0.056, SD = 0.236). *R*^2^Marginal = 0.102, *R*^2^Conditional = 0.561. Est., estimate; SE, standard error; OR, odds ratio; CI, confidence interval.

**Table 2:** Model summary of Fragmented Picture Task (FPT) model assessing implicit learning in all four FPT repetitions (*RT*~Repetition*Age).

Parameter	Est.	SE	*t*	*p*	*95% CI*
(Intercept)	7020.324	996.812			5066.607; 8974.041
Rep 1 to 2	–901.600	754.337	–1.195	0.241	–2380.072; 576.873
Rep 1 to 3	–3561.333	754.524	–4.720	<**0.001**	–5040.173; –2082.494
Rep 1 to 4	–2924.402	549.957	–5.318	<**0.001**	–4002.298; –1846.506
Age	16.126	17.457	0.924	0.357	–18.089; 50.342
Rep 1 to 2 * Age	9.555	6.263	1.526	0.127	–2.721; 21.830
Rep 1 to 3 * Age	5.603	6.269	0.894	0.372	–6.684; 17.889
Rep 1 to 4 * Age	–6.406	6.270	–1.022	0.307	–18.695; 5.884

Response times in this model were not transformed as normality of residuals was not improved with transformations. Repetitions were contrast coded such that each repetition was compared to the first presentation of the FPT. Values meeting the significance threshold of α < 0.05 are marked in **bold**. This model included random intercepts for participant (σ = > 10 m, SD = 3,183) and item (σ = > 4 m, SD = 2,066). *R*^2^Marginal = 0.381, *R*^2^Conditional = 0.697. Est., estimate; SE, standard error; CI, confidence interval. No Odds Ratios were computed due to the untransformed nature of the data.

On the SRT, response times (RTs) decreased reliably from block 1 to 4 (β = –0.217, *t* = –5.842, *p* < 0.001, 95% CI [–0.288; –0.143]) and block 1 to 5 (β = –0.314, *t* = –8.465, *p* < 0.001, 95% CI [–0.386; –0.241]), indicating successful learning of the SRT sequence. No overall age-related slowing was observed (*p* > 0.05); RTs did suggest the magnitude of facilitation between block 1 and 4 and between block 1 and 5 reduced with greater age, but effects were small (block 4 β = 0.001, *t* = 2.408, *p* < 0.05, 95% CI [0.000; 0.002]; block 5 β = 0.001, *t* = 3.050, 95% CI [0.000; 0.002]). On the FPT, RTs similarly decreased robustly between the first and third presentations (β = –3,561, *t* = –4.720, *p* < 0.001, 95% CI [–5,040; –2,082]) and the first and fourth presentations (β = –2,924, *t* = –5.318, *p* < 0.001, 95% CI [–4,002; –1,846]). No age effects appeared anywhere on the FPT (all *p*s > 0.05), which was confirmed by our Bayesian model comparisons (BFRep * Age v. Rep + Age < 0.000).

### 3.2 Structural priming task

Our second aim was to investigate the role of implicit and explicit memory in language processing. The structural priming task in this study presented active and passive sentence-picture matching trials (targets), preceded either by active or passive picture verification prompts (primes), thus creating a 2 × 2 design. We assessed target accuracy by prime structure, as well as by implicit and explicit composite scores (IS and ES). Figure 2 visualizes accuracy by prime and target type, showing clear differences between prime conditions on passives, but possibly not on actives. Table 3 therefore summarizes only the passive–only models. The models including both structures can be found in our supplementary materials at https://osf.io/4mw5x.

**Table 3:** Model summaries of structural priming sentence-picture matching generalized linear models predicting accuracy by prime structure for passive targets only, including either composite implicit scores (*Accuracy*~Prime*Age*IS), explicit scores (*Accuracy*~Prime*Age*ES), or neither (*Accuracy*~Prime*Age).

Model	Parameter	Est.	SE	*t*	*p*	*OR*	*95% CI*
Neither	(Intercept)	0.578	0.176				0.235; 0.923
Score	Prime: passive	0.533	0.266	2.004	**0.045**	1.704	0.012; 1.056
	Age	0.002	0.003	0.519	0.604	1.002	–0.005; 0.008
	Prime: passive * Age	0.001	0.005	0.261	0.794	1.001	–0.009; 0.012
IS	(Intercept)	00.583	0.1818				0.228; 0.941
	Prime: passive	0.571	0.2753	2.075	**0.038**	1.770	0.032; 1.112
	IS	–0.106	0.2133	–0.496	0.620	0.900	–0.523; 0.315
	Age	0.002	0.0043	0.500	0.617	1.002	–0.005; 0.009
	Prime: passive * IS	0.295	0.326	0.905	0.366	1.343	–0.340; 0.940
	Prime: passive * Age	–0.000	0.006	–0.010	0.992	1.000	–0.011; 0.011
	IS * Age	0.003	0.007	0.377	0.706	1.003	–0.012; 0.0171
	Prime: passive * IS * Age	–0.010	0.011	–0.928	0.353	0.990	–0.032; 0.011
ES	(Intercept)	0.566	0.179				0.216; 0.919
	Prime: passive	0.565	0.272	2.081	**0.037**	1.760	0.034; 1.099
	ES	–0.129	0.192	–0.673	0.501	0.879	–0.505; 0.247
	Age	0.002	0.004	0.622	0.534	1.002	-0.005; 0.009
	Prime: passive * ES	0.347	0.294	1.179	0.238	1.414	–0.229; 0.924
	Prime: passive * Age	0.000	0.005	0.048	0.962	1.000	–0.010; 0.011
	ES * Age	0.003	0.004	0.758	0.448	1.003	–0.004; 0.010
	Prime: passive * ES * Age	–0.008	0.006	–1.346	0.178	0.992	–0.019; 0.003

Values meeting the significance threshold of α < 0.05 are marked in **bold**. These models did not converge with random intercepts. *R*^2^No Scores = 0.018, *R*^2^IS = 0.018, *R*^2^ES = 0.019. Est., estimate; SE, standard error; OR, Odds Ratio; CI, confidence interval.

All models showed robust priming effects: accuracy was higher on passives when preceded by passive primes in the model not including either score (β = 0.533, *t* = 2.004, *p* < 0.05, 95% CI [0.012; 1.056]), the IS model (β = 0.571, *t* = 2.075, *p* < 0.05, 95% CI [0.032; 1.112]), and the ES model (β = 0.565, *t* = 2.081, *p* < 0.05, 95% CI [0.034; 1.099]). Structural priming was therefore achieved. Priming was further unaffected by age (all main age effect and interaction *p*s > 0.05), which was further confirmed with Bayesian model comparisons (BFPrime * Age v. Prime + Age = 0.014). Crucially, neither IS (β = –0.106, *t* = –0.496, *p* > 0.05, 95% CI [–0.523; 0.315]) nor ES (β = –0.129, *t* = –0.673, *p* > 0.05, 95% CI [–0.505; 2.47]) affected accuracy on this task, and neither score affected the strength of priming effects (Prime * IS interaction β = –0.010, *t* = –0.928, *p* > 0.05, 95% CI [–0.032; 0.011]; Prime * ES interaction β = –0.008, *t* = –1.346, *p* > 0.05, 95% CI [–0.019; 0.003]). Bayesian models also confirmed the absence of IS and ES effects on priming (BFIS * Prime v IS + Prime = 0.226; BF ES * Prime v ES + Prime = 0.227).

## 4 Discussion

This study aimed to assess implicit and explicit memory across the lifespan and the relative contribution of each memory system to language processing. A large sample of age-continuous participants completed a set of implicit and explicit memory tasks before participating in a structural priming sentence-picture matching task. Performance on implicit memory measures was assessed in detail, and composite explicit and implicit memory scores were related to priming task results.

### 4.1 Memory and aging

The current results are suggestive of age-related declines in explicit memory abilities, in line with substantial previous research (e.g., Park and Festini, 2017; Bäckman et al., 2001). Age correlated robustly with composite explicit memory scores. However, age did not correlate with composite implicit memory scores (see Figure 6), suggesting, at least at a surface level, that age does not affect implicit memory (in line with Reber et al., 1999; Rieckmann and Bäckman, 2009). More detailed analyses of our implicit memory measures nevertheless complicate this notion. Results from the implicit memory tasks included in this study (SRT and FPT) yield a multi-faceted picture. On the one hand, performance on the FPT remained stable across the lifespan, with age not affecting response times across task repetitions or interacting with each repetition separately. The core process of implicit FPT sequence learning therefore seems unaffected by age. However, fine-grained analyses of the SRT results are suggestive of age-related differences. Older adults showed somewhat reduced learning of the SRT sequence from block 1 to 4, and from block 1 to 5, although effect sizes were small.

We therefore cannot conclude that implicit learning remains unaffected by age, although the magnitude of our effects was small. Instead, we cautiously align our results with the framework of (Howard and Howard 2022), who make the case for fine-grained, subtle reductions in implicit learning efficacy with greater age. We further agree that such impairments are possibly difficult to uncover: the overall composite implicit learning scores we computed did not correlate with age, and performance on the FPT was also age-invariant. Only on fine-grained, detailed measures in our SRT data were age-related changes uncovered. Our data do, however, disagree with (Howard and Howard 2022)'s account in one important way: while (Howard and Howard 2022) highlight probabilistic sequence learning as the locus of age-related implicit memory impairments, we found declines on a non-probabilistic, linear sequence learning task. Thus, while many past studies finding age-related implicit memory changes have had to resort to using fairly complicated tasks (involving, for instance, third order sequential learning, see Bennett et al., 2007), we found evidence of declines on a comparatively simple task. Age-related impairments may well occur on such complex tasks, and on probabilistic sequence learning tasks, but they may therefore also appear on simpler, linear implicit memory tasks. Our evidence also disagrees with some past studies (e.g., Simon et al., 2012; King et al., 2013) which suggest age-related changes to implicit memory are not found in early stages of learning, but predominantly in later stages. Indeed, we found age-related declines only in the initial acquisition of the SRT sequence, while rebound effects were not sigificantly different with age.

The modality of our SRT task may be responsible for at least some of these effects. Many different variants of SRTs have been used in past research, from non-verbal (e.g., Bo et al., 2011), to visuospatial (e.g., Lum et al., 2019) to alternating SRTs (Howard and Howard, 2013) to formats closer to our own (Schuchard and Thompson, 2014). Notably, the SRT used in this study combined audio and visual input (as the task was initially designed for use in individuals with aphasia), unlike many other SRTs used in past research. It is possible this format taxes cognition somewhat differently to others', though we make no specific suggestions or hypotheses about the necessary skills underlying successful performance on our task. Given the absence of age-related declines on FPT performance and a correlation between age and composite implicit memory score, the question is raised whether the age-related declines on the SRT are indeed reflective of age effects or of substantial inter-participant variability or small performance differences that may not bear substantial theoretical impact. While this is not a possibility we can discount entirely, as shown in Figure 7 and Table 1 the standard errors associated with the SRT were not disproportionately high, and we accounted for inter-participant variability using a random intercept in the SRT model. We are therefore at least cautiously confident our results were not tainted by inter-individual variability to an extent to make overall pattern deduction impossible.

In short, our first research question (whether fine-grained implicit memory operations decline across the adult lifespan, and whether such declines mirror explicit memory impairments) is therefore answered partially—and tentatively—affirmatively. We did uncover possible fine-grained implicit memory declines in older adults, though effects were small and not comparable to declines observed in explicit memory measures.

### 4.2 Memory for language across the lifespan

Our second aim was to relate changing implicit and explicit memory skills across the lifespan with linguistic processing. Whether language processing declines with greater age is still a topic of significant controversy in current research (e.g., Poulisse et al., 2019; Van Boxtel and Lawyer, 2023a,b; Hardy et al., 2017, 2020), and it is unclear how implicit memory skills interact with language-related processing in older adults. The linguistic task used in this study attempted to elicit structural priming effects (that is, processing facilitations following from experience processing specific grammatical structures, in our case passives; see Ferreira and Bock, 2006, for a review). Structural priming is thought to rely, at least partially, on implicit learning (Heyselaar et al., 2021), and is therefore a prime candidate for assessing how implicit memory interacts with language processing across the lifespan.

Across ages, we found reliable effects of structural priming, such that passive sentence-picture matching trials showed higher accuracy when preceded by passive rather than active picture verification primes. These findings align with substantial past literature on priming in younger (Branigan, 2007; Tooley and Traxler, 2010; Mahowald et al., 2016) and older populations (Hardy et al., 2017, 2020; Van Boxtel and Lawyer, 2023a,b). We further concur with past notions (e.g., by Chang et al., 2006; Heyselaar et al., 2021) that priming does not rely on explicit memory abilities: we found no relations between priming effects and explicit memory scores across ages or in interactions with age.

However, we also did not find such relations between priming and implicit memory scores. Relating implicit memory test scores to priming has proven problematic in many past studies (e.g., Van Boxtel and Lawyer, 2023b), and even the recent suggestion by (Heyselaar et al. 2021) that different components of implicit memory subserve priming across the lifespan was not backed with direct relations of implicit memory to priming, but rather by varying different memory demands. We do not disagree that priming appears to be an effect driven by implicit adaptation to interlocutors—strong evidence from cumulative and conversational priming is convincing in this area (Reitter and Moore, 2006; Jaeger and Snider, 2013; Kaschak et al., 2014; Mahowald et al., 2016). Nevertheless, to our knowledge, no one measure of implicit memory reliably indexes the same (or similar) cognitive skills as are tapped by structural priming, leaving unanswered the critical issue of on what cognitive skills structural priming really relies.

Given this, and given past studies' difficulty establishing reliable relations between structural priming effects and cognitive tests, our results raise the possibility that priming is not reflective of domain-general learning mechanisms, but rather of some language-specific adaptation process that is not directly related to implicit memory. Indeed, some past theoretical accounts of structural priming suggest that priming-related facilitation relies on language-specific functions, such as structural prediction (e.g., Jaeger and Snider, 2013; Yang et al., 2021), lexical access (e.g., Reitter et al., 2011), or even the classic “spreading activation” proposed by many early priming studies (e.g., Pickering and Branigan, 1999), rather than (primarily) on domain-general implicit learning. The current results are at least indirectly supportive of these accounts, which may explain why no associations were found between implicit learning and the strength of priming effects in this study, and perhaps some previous studies.

Spreading activation theories of structural priming, grounded in distributional theories of semantic access and processing (e.g., Rumelhart et al., 1986; McRae et al., 2002), suggest the retrieval of a particular semantic cue raises the activation threshold of that cue, but also, critically, of surrounding and related cues. Cues distributed closely in semantic networks, when activated, spread activation to other cues in the same network, leading to priming effects on subsequent retrieval attempts. Such spreading activation models of priming (see Pickering and Branigan, 1999, for a salient example) have largely fallen out of fashion as implicit learning and dual-mechanism accounts were formulated which seem to more accurately model important tenents of structural priming, such as cumulative priming (Kaschak et al., 2014) and inverse frequency effects (e.g., Jaeger and Snider, 2013). While the present study does not aim to discount the important role of gradual, cumulative adaptation of the language processing system as foundational to priming effects, we do raise the absence of empirical connections between priming effects and measures of implicit learning as a highly critical shortcoming in implicit learning accounts of priming, and one which such accounts must explain in future research.

Methodologically, the specific format of our priming task may be at least partially responsible for the absence of statistical relations with memory scores. While structural priming is indisputably an implicit process (see above), our priming task may have required the recruitment of some explicit resources: the oral repetition of prime sentences and forced-choice picture matching may have muddled the boundary between implicit and explicit processes to such an extent that relations with our implicit and explicit memory measures became impossible. This nevertheless raises the question why similar task formats have yielded significant relations with memory measures in past studies (e.g., Man, 2023). This topic necessarily deserves substantial attention in future research.

Finally, the possibility exists that processing speed declines across the adult lifespan were at least somewhat influential in this study. Speed declines are a robust facet of the aging process (e.g., Salthouse, 1979, 1996; Finkel et al., 2007, among many others) and may be (partially) responsible for many observed age-related declines on linguistic tasks, especially those relying on explicit recall (see Van Boxtel and Lawyer, 2021, for a discussion). While we cannot fully discount the role of processing speed in the current study, as we did not record a measure of processing speed to use as a covariate or statistical control, previous findings from our group are less suggestive of processing speed limitations affecting structural priming specifically. Both (Van Boxtel and Lawyer 2023a) and (Van Boxtel and Lawyer 2023b) recorded structural priming in older and younger adults and used a measure of processing speed (the Letter Comparison Task) to predict priming effects. Speed scores were generally not predictive of priming effects in either study, excepting a small interaction in which greater processing speed was associated with somewhat greater priming, but crucially this effect did not interact with age (Van Boxtel and Lawyer, 2023a). We thus deem it unlikely—though not impossible—that processing speed differences formed a substantial confound in this study.

In short, our second research question (on how implicit memory relates to language processing across the lifespan and whether older adults rely differently on implicit memory during language processing than younger adults) cannot be definitively answered with our present data. This study does not find any relations between implicit or explicit memory performance and language processing (at least, as indexed by the structural priming task), and cannot claim older adults rely on implicit memory differently to younger adults when processing language.

### 4.3 Limitations and future directions

While this study thus makes important contributions to theoretical knowledge on the interplay between language, memory, and aging, we nevertheless acknowledge a number of limitations inherent in this study, and make recommendations for future research. First, although a comparatively large sample of participants was included, and statistical power was adequate, no participants over 80 years old were part of our sample, and age ranges were not distributed perfectly equally (see Figure 1. This may have played a role in the small effect sizes we found in our analyis of the SRT data. Relatedly, this study was run online rather than in person, and while we were careful in eliminating participants who may not have been paying close attention, we were less able to control for attentiveness than we may have been in person. Of all commonly used online participant recruitment platforms, Prolific.com—the platform used here—does appear to yield the most reliable data (Peer et al., 2022), but worthwhile efforts may include replicating the current findings with in-person participants.

Second, we acknowledge some limitations pertaining to our structural priming task, and whether it truly ‘isolated' implicit linguistic functioning from explicit cognition. Given that implicit and explicit abilities appear to exist along a continuum of awareness (see the introduction for a discussion) finding any one task which *only* taps implicit functions may be a tall order; however, we nevertheless suggest structural priming tasks do predominantly rely on implicit memory, and that our specific task format did reliably assess implicit linguistic functioning. While possible explicit demands during the sentence repetition stage of our priming task is a confound we acknowledge, priming effects themselves are well-established as predominantly linguistic (see, e.g., Ferreira and Bock, 2006; Chang et al., 2006).

Finally, the structural priming task we used necessarily indexed only certain sub-components of language processing, which is in itself a highly complex and multifaceted process. While we are convinced structural priming reflects important aspects of linguistic processing (a conviction backed by substantial amounts of past research, including but not limited to studies from our own group, e.g., Ferreira and Bock, 2006; Mahowald et al., 2016; Hardy et al., 2017, 2020; Heyselaar et al., 2021; Van Boxtel and Lawyer, 2023a,b) it does not encapsulate all stages of, and constraints affecting, such processing. It will be important for future studies to use related linguistic paradigms which tap processing quality and specific processing strategies, such as disambiguation paradigms (Van Boxtel and Lawyer, 2024), grammaticality judgements (Poulisse et al., 2019), and other tasks.

## 5 Conclusions

In short, the current study makes the case for viewing implicit learning as less affected by the aging process than explicit memory, but not unaffected. Fine-grained implicit memory impairments were found in older participants, but these did not appear to affect the efficacy of language processing. While composite implicit memory scores did not relate to age, explicit scores did, with substantial declines in explicit memory found in older participants. Across the lifespan, structural priming remains effective in spite of memory declines, suggesting older adults successfully leverage intact language processing, or efficient compensation skills, to achieve effective processing of linguistic input.

## Data Availability

The datasets presented in this study can be found in online repositories. The names of the repository/repositories and accession number(s) can be found below: https://osf.io/4mw5x.
